# Methodological evaluation of original articles on radiomics and machine learning for outcome prediction based on positron emission tomography (PET)

**DOI:** 10.1055/a-2198-0545

**Published:** 2023-11-23

**Authors:** Julian Manuel Michael Rogasch, Kuangyu Shi, David Kersting, Robert Seifert

**Affiliations:** 1 Department of Nuclear Medicine, Charité – Universitätsmedizin Berlin, corporate member of Freie Universität Berlin and Humboldt-Universität zu Berlin, Berlin, Germany; 2 Berlin Institute of Health at Charité – Universitätsmedizin Berlin, Berlin; 3 27252 Department of Nuclear Medicine, Inselspital University Hospital Bern, Bern, Switzerland; 4 39081 Department of Nuclear Medicine, University Hospital Essen, Essen, Germany

**Keywords:** radiomics, positron emission tomography, artificial intelligence, machine learning, TRIPOD, outcome prediction

## Abstract

**Aim** Despite a vast number of articles on radiomics and machine learning in positron emission tomography (PET) imaging, clinical applicability remains limited, partly owing to poor methodological quality. We therefore systematically investigated the methodology described in publications on radiomics and machine learning for PET-based outcome prediction.

**Methods** A systematic search for original articles was run on PubMed. All articles were rated according to 17 criteria proposed by the authors. Criteria with >2 rating categories were binarized into “adequate” or “inadequate”. The association between the number of “adequate” criteria per article and the date of publication was examined.

**Results** One hundred articles were identified (published between 07/2017 and 09/2023). The median proportion of articles per criterion that were rated “adequate” was 65% (range: 23–98%). Nineteen articles (19%) mentioned neither a test cohort nor cross-validation to separate training from testing. The median number of criteria with an “adequate” rating per article was 12.5 out of 17 (range, 4–17), and this did not increase with later dates of publication (Spearman’s rho, 0.094; p = 0.35). In 22 articles (22%), less than half of the items were rated “adequate”. Only 8% of articles published the source code, and 10% made the dataset openly available.

**Conclusion** Among the articles investigated, methodological weaknesses have been identified, and the degree of compliance with recommendations on methodological quality and reporting shows potential for improvement. Better adherence to established guidelines could increase the clinical significance of radiomics and machine learning for PET-based outcome prediction and finally lead to the widespread use in routine clinical practice.

## Introduction

In addition to its clinical value for tumor detection and staging, metabolic and/or molecular information derived from positron emission tomography (PET) imaging can facilitate the prognostication of survival and the prediction of treatment outcomes in various tumor types 1 2 3 . Traditionally employed image-derived features comprise standardized uptake values (SUV) and metabolic tumor volume (MTV) as well as composite metrics like total lesion glycolysis. These metrics are most commonly derived from manually or semi-automatically delineated regions of interest. In recent years, radiomics and machine learning-based prediction models have been increasingly employed to enhance the prognostic or predictive value of PET imaging by leveraging textural information and patterns that are not directly accessible to human readers 4 5 . However, the increasing complexity and feature number of such approaches, compared to the sparsity of manually derived image features, brings with it a higher risk of obtaining results that are either biased or not reproducible. Finally, despite the variety of prognostic radiomics and machine learning-based models published so far, broad clinical applicability has still not been achieved.

Different guidelines and recommendations have been published to define standards for radiomics and multivariable prediction approaches 6 7 8 . A common characteristic of these guidelines is that they propose standard practices related to both methodological quality and the transparency of reporting. This is a necessity, as readers, journal editors and reviewers of radiomics and machine learning articles should be enabled to fully assess the methodological approach. Ideally, a full description of methodological details should enable readers to repeat the experiment themselves. For example, the TRIPOD (Transparent Reporting of a multivariable prediction model for Individual Prognosis Or Diagnosis) statement has set standards for categorizing studies on multivariable models based on the type of internal or external validation that was used. The European Association of Nuclear Medicine (EANM) and Society of Nuclear Medicine and Molecular Imaging (SNMMI) have recently published joint recommendations on radiomics research in nuclear medicine 6 . Lambin *et al.* have proposed a radiomics quality score (RQS) to assess the methodological quality of radiomics studies 7 .

Using the TRIPOD criteria and RQS, Park *et al.* rated 77 radiomics studies in oncology and found that at 9.4, the mean RQS was only 26% of the maximum score of 36. Furthermore, TRIPOD recommendations were followed to a varying degree (2.6% to 100%). However, only 4% of the evaluated articles investigated PET, and only 20% of the studies focused on prognostic tasks while the majority assessed diagnostic applications 9 .

The RQS defines several aspects that should be considered in radiomics analyses but does not rate or quantify the appropriateness of the implementation of methods. TRIPOD recommendations are primarily focused on the transparency of reporting rather than provision of methodological guidance and are not specifically optimized for radiomics analyses.

On this basis, we set out to further explore the methodological approaches and reporting transparency of radiomics and machine learning articles that focus on PET-based outcome prediction using our own set of objective and subjective rating criteria. Our assessment was designed to resemble that of independent reviewers, journal editors or readers who would critically judge not only the concordance with specific methodological aspects but also the overall appropriateness of methods in the individual study context and the overall reliability of results and conclusions. Furthermore, we examined whether ratings improved with later dates of publication.

## Material and Methods

### Search strategy

Original articles were identified by a single person (JMMR) through a search on PubMed on July 15 and 16, 2023. Search prompts and results are given in Table 1 . Only original articles in English that investigated outcome prediction with PET using radiomics / textural features and/or machine learning methods (including neural networks) for classification/regression and/or to extract image features were considered for this analysis. Articles that investigated only a classification task (e.g., ”predicting” histological subtypes) were not considered eligible. Date of publication was not restricted.

**Table:** **Table 1** Search prompts and total number of article results.

Search prompt	Number of articles
(artificial intelligence[Title/Abstract]) AND (PET[Title/Abstract]) AND (outcome[Title/Abstract]) AND (prediction[Title/Abstract])	12
(neural network[Title/Abstract]) AND (PET[Title/Abstract]) AND (outcome[Title/Abstract]) AND (prediction[Title/Abstract])	10
(automated[Title/Abstract]) AND (PET[Title/Abstract]) AND (outcome[Title/Abstract]) AND (prediction[Title/Abstract])	13
(neural network[Title/Abstract]) AND (PET[Title/Abstract]) AND (response[Title/Abstract]) AND (prediction[Title/Abstract])	8
neural network PSMA prediction	6
deep learning PSMA prediction	6
(neural network[Title/Abstract]) AND (PET[Title/Abstract]) AND (survival[Title/Abstract])	36
(artificial intelligence[Title/Abstract]) AND (PET[Title/Abstract]) AND (survival[Title/Abstract])	38
(radiomics[Title/Abstract]) AND (PET[Title/Abstract]) AND (outcome[Title/Abstract])	155

### Rating criteria and rating process

Rating criteria and their categories / scales were created by JMMR and RS in consensus based on the RQS 7 and TRIPOD recommendations 8 . A final set of seventeen criteria was drawn up ( Table 2 ). Some criteria were designed as binary items (e.g., whether a separate test cohort was analyzed). Other, more subjective items were rated on a 3- or 4-point scale. To facilitate the interpretation of the results, all rating criteria were binarized into “adequate” or “inadequate” rating categories ( Table 2 ). A description of how individual categories for each item were defined can be found in the **Supplemental Material** .

**Table:** **Table 2** List of rating criteria, their categories and the percentage of articles rated as “adequate” after binarization of the rating categories. Details on the precise definitions of all categories can be found in the **Supplemental Material** .

Rating criteria	Categories				Adequate articles (%)
	Adequate		Inadequate		
**Validity of the results**					
Homogeneous patient cohort?	Yes	Rather yes	Rather no	No	64
Presence of selection bias?	No	Rather no	Rather yes	Yes	88
Is the frequency of classes balanced?	Yes	Rather yes	Rather no	No	65
Training and testing split consistently (to prevent data leakage)?	Yes	Rather yes	Rather no	No	64
Clearly defined research question(s)?	Yes	Rather yes	Rather no	No	98
Clear main/primary endpoint for model training?	Yes	Rather yes	Rather no	No	86
Adequate statistical method for the primary endpoint?	Yes	Rather yes	Rather no	No	77
Comparison with established biomarkers?	Yes		No		65
Machine learning description informative?	Yes	Rather yes	Rather no	No	64
**Generalizability of the results**					
Risk of overfitting?	Low	Average	High		62
Separate test cohort / cross-validation present?	Yes		No		81
Robustness of results reported (e.g., resampling or confidence interval)?	Yes		No		39
Independent/external cohort used for testing?	Yes		No		23
**Results and conclusion**					
Is the presentation of results comprehensible?	Yes	Rather yes	Rather no	No	90
Are the results informative?	Yes	Rather yes	Rather no	No	72
Adequate conclusion with regard to validity?	Yes	Rather yes	Rather no	No	73
Adequate conclusion with regard to generalizability?	Yes	Rather yes	Rather no	No	59

A single rater (JMMR) assessed all original articles based on the seventeen criteria. If a certain criterion could not be rated (because the article did not contain the required information), it was rated as “unclear” and was also regarded as “inadequate”. Furthermore, the sample size of the training cohort/folds and test cohort/folds was noted and whether data collection included prospective or multicentric data. Whether the dataset and/or the machine learning code had been published on a public repository was also evaluated. The TRIPOD study type was identified, based on the methodology applied in the articles. On average, around 20 minutes were required to rate an article.

### Statistical analysis

Based on the Shapiro-Wilk test, a non-normal data distribution was assumed, and the median, interquartile range (IQR), and range were presented. The association between the date of publication and number of criteria with an “adequate” rating per article was analyzed by Spearman correlation using SPSS version 29.0.0.0 (IBM Corporation, Armond, NY, USA). Statistical significance was assumed at α = 0.05.

## Results

### Description of the original articles

A total of 107 original articles were identified through the search process. Three of these were excluded as no full text version could be accessed. One publication was excluded because it investigated only one single PET feature, while another article was not included because the declared aim was solely to validate a previously published model. Two publications examining PET in the context of Alzheimer's disease or amyotrophic lateral sclerosis were excluded, as these differed fundamentally from all the other articles where the focus was on oncology.

The remaining articles (n = 100), published between July 2017 and September 2023, were included in the subsequent analysis ( Table 3 ). The cancer entities investigated comprised head and neck cancer (n=26) 10 11 12 13 14 15 16 17 18 19 20 21 22 23 24 25 26 27 28 29 30 31 32 33 34 35 , lung cancer (n=20) 36 37 38 39 40 41 42 43 44 45 46 47 48 49 50 51 52 53 54 55 , gastrointestinal or hepatobiliary tumors (n=15) 56 57 58 59 60 61 62 63 64 65 66 67 68 69 70 , lymphoma (n=13) 3 71 72 73 74 75 76 77 78 79 80 81 82 , gynecological tumors (n=7) 83 84 85 86 87 88 89 , prostate cancer (n=5) 90 91 92 93 94 , breast cancer (n=3) 95 96 97 , brain tumors (n=3) 98 99 100 , sarcoma (n=2) 101 102 , melanoma (n=2) 103 104 , multiple myeloma (n=2) 105 106 , salivary gland tumors (n=1) 107 and pleural mesothelioma (n=1) 108 , respectively.

**Table:** **Table 3** General characteristics of the articles analyzed.

Characteristics	Number of articles
Total	100
Type of image analysis (categories based on 6 )	
Only hand-crafted radiomics	50
Radiomics and machine learning for prediction (“hybrid radiomics”)	46
Only machine learning for prediction	4
Year of publication	
2017	1
2018	7
2019	13
2020	15
2021	20
2022	29
2023	15
Prospective data included	19
Multicentric data included	30
TRIPOD study type	
1a	15
1b	26
2a	31
2b	8
3	16
Unclear (considered equivalent to type 1a)	4

The median sample size of the training cohort/folds was 86 (IQR, 49–172; range, 20–1049). Nineteen articles (19%) reported neither a test cohort nor cross-validation to separate training from testing data. In the other 81 articles, the median sample size of the test cohort (fold in case of cross-validation) was 40 (IQR, 20–85; range, 1–887). A test sample size of one in two articles was due to bootstrapping. This is considered adequate by the TRIPOD recommendations (study type 1b).

In 8 articles (8%), the machine learning code was published, while in 10 (10%) the datasets were made publically available.

### Rating results

The median proportion of articles per criterion with “adequate” rating was 65% (IQR, 63–84), ranging from 23% (criterion: “Independent/external cohort used for testing?”) to 98% (criterion: “Clearly defined research question(s)?”).

**Fig. 1:**
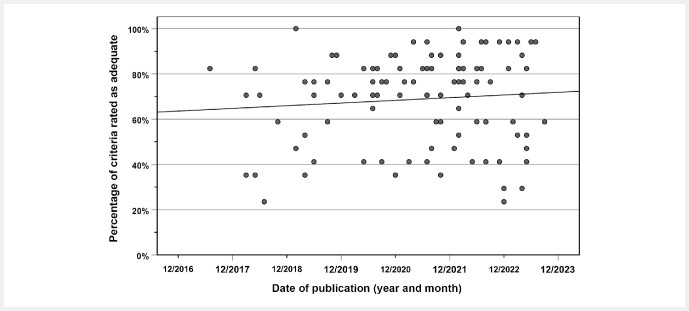
Correlation between date of publication (grid: months) and percentage of criteria per article that were rated as “adequate” (the maximum of 17 criteria would correspond to 100%). Solid line: linear fit.

The median number of criteria with an “adequate” rating per article was 12.5 (IQR, 9–14; range, 4–17). Two articles 12 42 were rated “adequate” in all 17 criteria, whereas 22 articles (22%) had an “adequate” rating in less than half of the items (≤8 of 17 items). The number of criteria with an “adequate” rating per article was not associated with the date of publication (Spearman’s rho, 0.094; p = 0.35; Fig. 1 ).

Detailed results item-by-item for all articles are available as open data in the Zenodo repository https://doi.org/10.5281/zenodo.8284734 .

## Discussion

The aim of this investigation was to analyze the appropriateness of methodological approaches and their description in studies on radiomics or machine learning analysis in PET-based outcome prediction. To this end, a multi-dimensional set of seventeen items was drawn up and applied to 100 articles retrieved from a systematic PubMed search. On average, we found that per criterion a median of 65% of articles achieved an “adequate” rating.

The fraction of 65% adequate articles per criterion based on the comprehensive review may seem high, but it should be noted that the criteria and rating categories used were designed to reflect scientific reality and are not an idealized situation. This is underlined by the observation that two articles 12 42 achieved an “adequate” rating for all 17 criteria assessed by us. Like us, Park *et al.* examined 77 articles on radiomics but reported that at 9.4, the average RQS was only 26.1% of the maximum of 36 9 . As we used different rating criteria, these results can only be compared at the level of individual items. Like that of Park *et al.,* our analysis showed that most of the studies reserved a separate cohort or used cross-validation for testing (here: 81%; Park *et al.* : 90%). However, it is worth noting that 19% did not do so, which means that the corresponding results are purely exploratory and cannot be generalized. Neither should they be applied in clinical routine practice without prior validation. Most articles used adequate statistical (discrimination) methods for the primary endpoint, such as area under the curve (AUC) or concordance index (77%). Park *et al.* reported even higher adherence (99%), probably because most studies analyzed by Park *et al.* were diagnostic studies that usually use binary endpoints and can rely on the AUC. We also found that resampling was employed to examine the robustness of results by only 39% of the studies examined (this was similar to Park *et al.:* 30%). Validation/testing with an independent (external) dataset was unfortunately not frequently observed (23%), and datasets or source codes were rarely made available (10 and 8%, respectively), which is in line with Park *et al* . (external validation: 18%; open science: 4%).

The TRIPOD statement and RQS provide a list of methods or report elements that should be observed. However, beyond simply following such a checklist of recommendations, authors should also report how rigorously the methods were implemented because simply using tools such as cross-validation or feature selection does not guarantee their proper implementation to completely prevent data leakage. We think that authors should make every effort to enable readers to understand the way that such crucial methodological steps were actually realized. Positive examples included articles that used flow charts to visualize the separation of training/test and other methodological steps. However, we found that key aspects were often not sufficiently covered by the articles (“informative” machine learning description in only 64% of articles).

One example of a RQS criterion that may – formally speaking – be fulfilled by many articles is the use of a multivariable analysis such as Cox regression. In many of the investigated articles, Cox regression was used for feature selection or to investigate the independent value of predictors. However, proper implementation requires that weights are kept unchanged between training and testing. Otherwise, retraining would invalidate the training/testing separation approach. Unfortunately, this was often not stated. In general, descriptions of machine learning methods are rarely given in sufficient detail (or supported by open data code) to allow other researchers to reliably reproduce the selected features and final results.

In studies with multiple endpoints (e.g., progression yes/no, progression-free survival, and overall survival), the authors should specify the endpoint used for training. If a binary endpoint was used, several of the investigated studies reported multiple performance metrics (e.g., AUC and accuracy) and omitted to state clearly, which of the mentioned performance metrics was used to gauge model training.

We identified aspects in methodology or reporting that are not (fully) covered by the TRIPOD statement or the RQS: the balance/imbalance of outcomes, an overall assessment of the risk of overfitting (in the light of sample size, feature selection methods, and loss of performance from training to testing) or the adequateness of conclusions. We strongly feel that these criteria are also important factors for judging the validity of radiomics results. The proposed set of criteria is therefore useful for reviewers of radiomics and machine learning articles for PET-based predictions.

In order to objectify our criteria, we have included descriptions of all subjective rating categories ( **Supplemental Material** ), which should enable readers to understand and interpret our findings. We also point out the limitations of our approach and state which standards were required for an “adequate” rating. We hope that these descriptions will improve the reproducibility of the rating. It is furthermore worth noting that a single person rated the articles, and subjective ratings may be influenced by personal expectations and demands on the quality of radiomics papers. Furthermore, inter-rater variability could not be determined. Our analysis and its transferability to other analyses is biased, because we used our own set of rating criteria and categories. However, our findings on specific items that are also part of the RQS were comparable to Park *et al.* 9 , indicating the validity of the single observer assessment at least for these criteria.

Regardless of the potential subjectivity of the evaluation criteria, the observation remains that the average rating, which is a measure of the quality of the paper, did not change (improve) over the time period 07/2017 to 09/2023. Notably, the TRIPOD statement was published in 2015 8 and the RQS in 2017 7 . Still, the impact of these recommendations on how radiomics and machine learning analyses are performed and reported in the context of outcome prediction with PET appears to be limited. Considering that the clinical applicability of radiomics analyses remains low, despite the large number of published models, improvements are clearly needed.

## Conclusion

The methodological quality and reporting transparency of the investigated papers on radiomics and machine learning for PET-based outcome prediction often appeared inadequate. Authors and reviewers of such articles should aim at enabling the reader to reproduce the results and to this end, certain critical quality criteria must be met (e.g., clear identification of event rates, the endpoint and primary performance metric used for model training, and a clear description of the separation of training/testing to identify potential data leakage). Better adherence to previously published recommendations is imperative to finally enable the widespread use of radiomics and machine learning in routine clinical practice.

## Supporting information

Supplementary material Zusatzmaterial
