# Supplementary material for: Methodological evaluation of original articles on radiomics and machine learning for outcome prediction based on positron emission tomography (PET)
Source: Nuklearmedizin. 2023 Nov 23;62(6):361–9. doi: 10.1055/a-2198-0545 (PMC10667066; doi:10.1055/a-2198-0545)
Supplement: Supplementary file 1 — Supplementary material [file 10-1055-a-2198-0545_22022472.pdf]

Supplemental Material

Article title: Methodological evaluation of original articles on radiomics and machine learning for outcome prediction based on positron emission tomography (PET)

Journal name: Nuklearmedizin

Author names: Julian M. M. Rogasch, Kuangyu Shi, David Kersting, Robert Seifert

Corresponding author:
Julian M. M. Rogasch
Charité – Universitätsmedizin Berlin
Department of Nuclear Medicine
Augustenburger Platz 1
D-13353 Berlin, Germany
Tel.: +49 30 450 627106
Fax: +49 30 450 7557338
E-Mail: julian.rogasch@charite.de
ORCID ID: 0000-0002-0817-6532

Full list of rating criteria and categories

Validity of the results:

Criteria and categories	Description
**Homogenous patient cohort?**	
Yes	e.g., single tumor type, confined to certain tumor stages or treatment
Rather yes	e.g., same tumor type and similar treatments
Rather no	e.g., different types of head and neck cancer, tumor stages I-IV or fundamentally different treatments
No	e.g., different tumor types
**Presence of selection bias?**	
No	Consecutive patient inclusion or broad inclusion criteria
Rather no	Uncritical exclusion criteria or clearly outlined patient selection (e.g., confined to a certain treatment or tumor stages or as part of a prospective study protocol)
Rather yes	Restrictive inclusion criteria that result in a potentially not representative cohort (e.g., availability of a follow-up PET/CT scan or rare PET tracer)
Yes	Patient selection appears arbitrary and opaque
**Is the frequency of classes balanced?**	
Yes	Minority class approx. 50% or slightly lower than 50% but adequate compensatory measures, e.g. oversampling
Rather yes	Minority class approx. 25-40% (± adequate performance metric for training, e.g., AUC instead of accuracy)
Rather no	Minority class approx. 10-25%
No	Minority class approx. ≤10%
**Training and testing split consistently (to prevent data leakage)?**	
Yes	Clear separation of training and test during all relevant steps (ideally illustrated by a flow diagram)
Rather yes	The methods description indicates adequate separation of training and testing

Rather no	The methods description indicates that training and testing were not adequately separated
No	Clear indications of improper separation of training and testing
**Clearly defined research question(s)?**	
Yes	Aim(s) and question(s) are stated clearly and comprehensibly
Rather yes	Aim(s) and question(s) are recognizable
Rather no	Aim(s) and question(s) are not readily recognizable or vague
No	Aim(s) and question(s) are not recognizable
**Clear main/primary endpoint for model training?**	
Yes	The endpoint used to train the model is clearly named.
Rather yes	The endpoint used for training is recognizable although not clearly named (e.g., if several endpoints are investigated, the reader can still identify the primary endpoint).
Rather no	The endpoint used for training cannot be clearly identified / remains vague.
No	The endpoint used for training cannot be identified at all.
**Adequate statistical method for the primary endpoint?**	
Yes	e.g., adequate performance metric and multivariable analysis for time-to-event data
Rather yes	e.g., adequate performance metric for binary endpoints
Rather no	e.g., lack of multivariable analysis although necessary
No	e.g., inadequate performance metric
**Comparison with established biomarkers?**	Yes/No
**Machine learning description informative?**	
Yes	All relevant aspects are described to an extent that may even allow other researchers to reproduce the results using the same dataset (including, for example,

	hyperparameters for the machine learning models).
Rather yes	All relevant aspects are described to an extent that allows readers to comprehend and assess the methods.
Rather no	Relevant aspects of the methods are either not described in sufficient detail or are not comprehensible.
No	Description of the methods does not fulfil even minimum requirements.

Generalizability of the results:

**Criteria and categories**	**Description**
**Risk of overfitting?**	
Low	Separate test cohort / cross-validation; feature selection; good ratio of final model variables to events; similar performance between training and test cohort/folds
Average	Separate test cohort / cross-validation; average to unfavourable ratio of model variables to events; tolerable performance discrepancy between training and test
High	No separate test cohort / cross-validation or lack of multivariable analysis or considerable loss of performance between training and test
**Separate test cohort / cross-validation present?**	Yes/No
**Robustness of results reported (e.g., resampling or confidence interval)?**	Yes (e.g., confidence interval from cross-validation or bootstrapping)/No
**Independent/external cohort used for testing?**	Yes (e.g., different scanner or center) / No

Results and conclusion:

**Criteria and categories**	**Description**
**Is the presentation of results comprehensible?**	
Yes	Readily comprehensible
Rather yes	Sufficiently comprehensible
Rather no	Rather difficult to understand
No	Incomprehensible
**Are the results informative?**	
Yes	Proportion of the minority class or number of events is given. Test performance is

	stated. Robustness of results is given. Comparison with other predictors is shown.
Rather yes	Three of the four above-named items are fulfilled.
Rather no	Only two of the four items are fulfilled.
No	Less than two of the four items are fulfilled.
**Adequate conclusion with regard to validity?**	
Yes	The conclusion on what the analysis has shown matches well the results and their certainty.
Rather yes	The conclusion is sufficiently supported by the validity/certainty of the results.
Rather no	The conclusion is not sufficiently supported by the results, e.g., because it does not account for a lack of statistical power.
No	The conclusion is clearly not supported by the results.
**Adequate conclusion with regard to generalizability?**	
Yes	The conclusion on how generalizable the results purportedly are matches well the results and how they were obtained from independent datasets or by an appropriate training/test strategy.
Rather yes	The conclusion is sufficiently supported by the generalizability of the results.
Rather no	The conclusion is not sufficiently supported by the generalizability of the results, e.g., because it does not account for relevant overfitting or lack of a sufficiently large test cohort/folds.
No	The conclusion is clearly not supported by the generalizability of the results.
